# Quinoa (*Chenopodium quinoa* Willd.): Genetic Diversity According to ISSR and SCoT Markers, Relative Gene Expression, and Morpho-Physiological Variation under Salinity Stress

**DOI:** 10.3390/plants10122802

**Published:** 2021-12-17

**Authors:** Diaa Abd El-Moneim, Eman I. S. ELsarag, Salman Aloufi, Asmaa M. El-Azraq, Salha Mesfer ALshamrani, Fatmah Ahmed Ahmed Safhi, Amira A. Ibrahim

**Affiliations:** 1 Department of Plant Production (Genetic Branch), Faculty of Environmental and Agricultural Sciences, Arish University, El-Arish 45511, Egypt; 2 Department of Plant Production (Agronomy Branch), Faculty of Environmental and Agricultural Sciences, Arish University, El-Arish 45511, Egypt; emanelsarag@aru.edu.eg (E.I.S.E.); asmaamoustafaalazrak@gmail.com (A.M.E.-A.); 3 Department of Biotechnology, Faculty of Science, Taif University, P.O. Box 11099, Taif 21944, Saudi Arabia; s.aloufi@tu.edu.sa; 4 Department of Biology, College of Science, University of Jeddah, Jeddah 21959, Saudi Arabia; smalshmrane@uj.edu.sa; 5 Department of Biology, College of Science, Princess Nourah bint Abdulrahman University, Riyadh 11671, Saudi Arabia; faalsafhi@pnu.edu.sa; 6 Plant Protection and Biomolecular Diagnosis Department, Arid Lands Cultivation Research Institute, City of Scientific Research and Technological Applications, New Borg El-Arab, Alexandria 21934, Egypt

**Keywords:** quinoa, salinity, morpho-physiological traits, chemical compositions, gene expression, *CqSOS1*, *CqNHX1*, ISSR, SCoT

## Abstract

Quinoa (*Chenopodium quinoa* Willd.) is a halophytic crop that can withstand a variety of abiotic stresses, including salt. The present research examined the mechanisms of salt tolerance in five different quinoa genotypes at four different salinity levels (control (60), 80, 120, and 160 mM NaCl). ISSR and SCoT analysis revealed high polymorphism percentages of 90.91% and 85.26%, respectively. Furthermore, ISSR 1 and SCoT 7 attained the greatest number of polymorphic amplicons (27 and 26), respectively. Notably, LINE-6 and M-28 genotypes demonstrated the greatest number of unique positive and negative amplicons (50 and 42) generated from ISSR and SCoT, respectively. Protein pattern analysis detected 11 bands with a polymorphism percentage 27.27% among the quinoa genotypes, with three unique bands distinguishable for the M-28 genotype. Similarity correlation indicated that the highest similarity was between S-10 and Regeolone-3 (0.657), while the lowest similarity was between M-28 and LINE-6 (0.44). Significant variations existed among the studied salinity treatments, genotypes, and the interactions between them. The highest and lowest values for all the studied morpho-physiological and biochemical traits were recorded at 60 and 160 mM NaCl concentrations, respectively, except for the Na and proline contents, which exhibited the opposite relationship. The M-28 genotype demonstrated the highest values for all studied characteristics, while the LINE-6 genotype represented the lowest in both seasons. On the other hand, mRNA transcript levels for *CqSOS1* did not exhibit differential expression in roots and leaf tissues, while the expression of *CqNHX1* was upregulated more in both tissues for the M-28 genotype than for the LINE-6 genotype, and its maximum induction was seen in the leaves. Overall, the genotypes M-28 and LINE-6 were identified as the most and least salinity-tolerant, respectively.

## 1. Introduction

Over recent decades, the world has experienced significant changes in climate and the subsequent impact on the growth, physiology, and yields of crops. In addition to climate change, short-sighted agricultural practices increased soil salinity in certain areas [1]. Quinoa (*Chenopodium quinoa* Willd.) belongs to the family Chenopodiacea and has been cultivated in different regions for over 7000 years due to its ability to adapt to various environmental conditions [2,3,4]. Recently, quinoa has gained global attention due to its high resistance to diverse abiotic stresses such as salinity, drought, those found in forest ecosystems, and heat [5]. It can be cultivated in different environments and geographical areas; as such, quinoa has become an alternative model crop in many marginal arid and semi-arid regions [3]. Its adaptability to various edaphic environmental stresses has led to its substantial genetic diversity, with more than 16,000 accessions stored in seed banks around the world [1]. There are five quinoa ecotypes according to the degree of adaptation to environmental conditions: Altiplano, Salar, Yunga, Valley, and Lowland [6,7].

Quinoa is a multipurpose plant, recently used in the human diet and as an alternative to animal products as a source of protein; quinoa contains high contents of protein (12–18%), amino acids, bioactive compounds, essential amino acids, fatty acids, and minerals [8,9]. The seeds and leaves of quinoa are used as food, while its biomass is also used in animal feed. Additionally, its high contents of saponins and colorants make it useful for industrial and pharmaceutical purposes [2,10]. Nevertheless, investigating the salt-tolerance capabilities of different quinoa genotypes and measuring their yield stability—without affecting grain quality—is critical to increasing the food security and agricultural productivity of resource-poor and degraded marginal lands. In this regard, estimation of the genetic diversity of quinoa will be helpful in the assessment of conservation and the development of core selections for breeding systems [11,12]. 

Genotype collection, assessment, and evaluation are essential steps in breeding programs [13]. The quinoa plant has characteristically large morphological properties that can help farmers in plant selection; however, this approach fails to provide genetic information that may restrict breeding strategies [14,15]. Salinity stress studies of quinoa are typically based on Na and K mineral contents due to their correlation with osmotic pathways and hydrophilic mechanisms [16,17,18]. Quinoa has a high mineral content (Na, K, Mg, Ca, and P) that researchers tend to focus on when studying plant breeding and the genetic diversity of this sustainable crop. 

Several genes encode different mechanisms implemented by salt-tolerant plants when apoplastic Na^+^ levels are high. Salt overly sensitive 1 (SOS1) can facilitate the loading of Na^+^ into xylem vessels from the roots, while NHX1 exchanges Na and H and leads to Na compartmentation in vacuoles [19]. An accumulation of sodium alters the K homeostasis that uses the Na/K ratio as an indicator for salinity tolerance in halophytes [20,21]. Physiological factors such as membrane transport systems, sodium uptake minimization, and ion compartmentation in cells and tissues are regulated by main genes such as SOS1 and NHX1 [22,23]. The analysis of gene expression levels and tissue specificity will help understand the mechanisms present in *C. quinoa*. It is possible that these genes also have a role in the molecular mechanisms that regulate salt responses in quinoa.

ISSRs (inter simple sequence repeats) markers are easy to use and repeat. It only needs a small amount of DNA and does not need to know the DNA sequence. ISSR primers are made from SSR motifs, and they can be used on any plant species with a suitable number and distribution of SSR motifs in its genome [24]. Furthermore, Start Codon Targeted (SCoT) polymorphisms are reproducible markers that are based on the short-conserved region in plant genes surrounding the ATG translation start codon; the use of SCoT markers would be much more efficient, especially in comparison to other arbitrary markers, due to the longer primer distances and high annealing temperatures [25]. The SCoT marker design approach does not require any detailed genomic sequence information, making it easier to apply to plants that do not have a reference genome [26]. Because of their high reproducibility and power for detecting polymorphism in quinoa, several investigations were concluded that both markers are helpful in genetic variability evaluation [27,28,29] using ISSR and [29,30] using SCoT. In the same context, SDS-PAGE protein profile analysis is a rapid method to detect and identify the relationships between different quinoa genotypes [31,32,33].

The lack of characterization and evaluation studies could limit selection and improvement as well as cultivation expansion. Hence, the present study will be of particular value in ongoing efforts to both accelerate the improvement of quinoa and develop core collections that can be used by traditional breeding programs in Egypt. This investigation aimed to (i) study the genetic diversity between studied quinoa genotypes using ISSR, SCoT, and SDS-PAGE protein analysis, (ii) study the physio-morphological and biochemical characters of quinoa genotypes under salinity stress, and (iii) evaluate the relative gene expression using *CqNHX1* and *CqSOS1* genes for the tolerant and sensitive genotypes.

## 2. Materials and Methods

### 2.1. Germplasm, Experimental Setup, and Growth Conditions

This research was conducted in a shading house at the Faculty of Environmental Agricultural Sciences, Arish University, North Sinai, Egypt, over two winter seasons (2016/2017 and 2017/2018). The names and sources of the genotypes are presented in Table 1. All the studied genotypes were imported by Desert Research Center, Egypt; then, through personal communication, we were granted to evaluate them under North Sinai conditions. For the whole experiment, 60 pots were used; 20 pots were planted with five genotypes (M-28, Q-37, S-10, Regeolone-3, Line-6; 10 seeds/pot) in 3 replications, then four salinity levels (control (60), 80, 120, and 160 mM NaCl) were applied for 60 pots after 20 days from planting. Plastic pots (15 cm diameter × 16 cm depth) were filled with a 3.00 kg mixture of sand and clay (1:1). After four days, seedlings were thinned at a rate of 24 seedlings/genotype (in three replicates; 8 seeds/pot). Phosphoric acid (H_2_PO_5_, 85%) and NPK (20:20:20) were added at rates of 1 cm/L and 1 g/L, respectively. The salinity level treatments were applied from 20 days after planting until three months later. The soil salinity was 4.46 dsm^−1^ before salinity levels application. The harvesting date was 110 days after the sowing date.

### 2.2. Measurements of Growth and Developmental Parameters

#### 2.2.1. Germination Rate

Grains were sterilized for 20 min in 20% NaOCl, rinsed, and soaked for one h in distilled water. The experiment was conducted at 25 ± 2 °C under dark conditions, and the germination was performed using saline water and filter paper in Petri dishes. Salinity treatments were 60, 80, 120, and 160 mM NaCl. Twenty-five seeds/genotype were sown in the Petri dishes with three replicates. The germinating seeds were regularly checked from three days after the sowing date. After 20 days, the germination rate (GR) was estimated according to Barlett [34].

#### 2.2.2. Morpho-Physiological Traits

Growth traits: Relative Growth Rate (RGR), Crop Growth Rate (CGR), Net Assimilation Rate (NAR)) were measured by collecting randomly three guarded plants from each pot at 45, 60, and 75 days after the sowing date and were computed according to Radford [35]. Physiological traits: Leaf Area (LA) (cm- dsm^2^) according to Radford [35] Leaf Area Index (LAI) according to Beedle [36], plant height (cm), root length (cm), number of leaves/plants, and number of branches/plants were recorded at 75 days after sowing date.

#### 2.2.3. Yield and Its Components

Grain weight (g/plant), Harvest Index (HI; %), 1000-grain weight (g), number of panicles/plants, total weight of panicles/plant (g), and plant fresh weight (g) were measured by collecting randomly five guarded plants from each pot at 110 days after sowing.

#### 2.2.4. Measurements of Chemical Compositions

Protein, proline, and mineral (N, P, K, and Na) contents were estimated by collecting samples at 60 days after sowing. Protein content: Seed meals were dried at 70 °C and kept for N analysis. Protein percentage was determined according to [37]. The formula used for calculation of crude protein was as follows: Protein% = (T × 0.1 × 14 × 100 × 6.2)/(Weight of sample × 1000) Where T: Transmittance. Proline content: 0.5 g leaves tissue was ground in 10 mL 3% Solphosalicylic acid using Bates method [38]. The solution was purified, and 2 mL was taken off from any solutions, then 2 mL ninhydrin acid agent and 2 mL acetic acid were added to the theme. Tubes soaked in Ban Mary for 1 h in 100 °C and then kept for 30 m in an ice bath, then 4 mL toluene added to tubes and two separate layers formed after shaking tubes and keeping them for 20 s. Finally, colored layer absorption during 520 nm waves and proline content were measured using a standard curve. Leaves mineral contents (N, P, K, and Na): leaves of 5 plants were dried at 70 °C for 48 h, and 0.5 g samples were digested by sulfuric acid and hydrogen peroxide to determine mineral content. According to [39]. After proper dilution of digested material, N was determined using the modified Kjeldahl method. Phosphorus was determined calorimetrically using a spectrophotometer. Potassium and Sodium were determined by using a flame photometer.

### 2.3. Molecular Characterization

#### 2.3.1. ISSR and SCoT Marker Analysis

##### DNA Extraction and ISSR and SCot Amplification

Young leaves of five quinoa genotypes were used for DNA extraction using the CTAB buffer protocol described by Cota-Sanchez et al. [40], and the concentration was determined using nanodrop. Ten pairs of primers from each ISSR and SCoT marker were used in this study. The names and sequences of the primers used are presented in Table S1. Amplification of DNA was carried out according to Zietkiewicz et al. [41] and Collard and Mackill [25] in 20 µL of PCR reaction mixture containing 10 µL of master (2X TOPsimple DyeMIX-nTaq), 5 µL of (0.1 μM) for each primer, and 1 µL of genomic DNA (50 ng/μL); the final volume of 20 µL was achieved using sterile distilled water. The reaction was performed on a Simple Applied Biosystems thermal cycler using the following thermal profile: pre-denaturing for 5 min at 95 °C, followed by 40 cycles at 94 °C for 40 s, annealing for 40 s, and 72 °C for 1 min, with a final incubation time of 5 min at 72 °C. The products were separated on a 1.5% agarose gel. Only clear, unambiguous, and reproducible bands are considered a single locus. Data were scored as (1) for presence and (0) for absence for each of five samples. If a band was absent in all the studied genotypes and appears in just one genotype, we consider it a positive, unique band. While if there is a band present in all genotypes and absent in just one genotype, we consider it a negative unique band. The polymorphism information content (PIC) was calculated according to the formula of [42], as follows: PIC = 1 − Σ*pi*^2^ where pi is the frequency of the *i*th allele of the locus in eight genotypes.

#### 2.3.2. Protein Analysis

Proteins were extracted from leaves of the studied genotypes using 20 mM Tris-Cl extraction buffer (pH 8.0) containing two mM EDTA and one mM PMSF. Protein concentration in each sample was determined according to Bradford [43]. SDS-PAGE (Sodium dodecyl sulphate–polyacrylamide gel electrophoresis) of the extracted Leaves protein was carried out on 15% polyacrylamide gel following the method of Laemmli [44]. The electrophoretic profile of leaves proteins of each genotype was recorded as presence (1) or absence (0) of a band of a particular molecular weight. The protein profile was analyzed using a Bio-Rad Gel Documentation System (BIO-RAD-Gel-DocModel2000).

#### 2.3.3. Gene Expression Analysis

Based on morpho-physiological analysis, two quinoa genotypes (M-28 and LINE-6) were chosen according to their contrasting salinity tolerances for the gene expression analysis. Ten germinated seeds were transferred into four 60 × 25 mm plastic boxes/genotype, with three replicates. The plastic boxes contained growth medium (Hoagland solution), which was changed daily. Seedlings were grown for seven days under normal conditions. On the eighth day, salt treatment was initiated by gradually adding NaCl to the Hoagland solution. Two boxes of each genotype (with three replicates) were treated with two treatments (control or 80 mM NaCl). After two days, the concentration of NaCl was increased to reach the final concentration of 160 mM NaCl. Overall, the salinity treatment lasted six days. After two days of exposure to the final concentration, plant leaves and roots were harvested and immediately frozen in liquid nitrogen and kept at −80 °C for further analysis. Subsequently, total RNA was extracted from 100 mg of leaves or roots using TRIzol reagent described by the manufacturer (Catalog 12183555, Invitrogen, Carlsbad, CA, USA). The mRNA levels of the studied genes were estimated using real-time PCR with an ABI Prism 7700 Sequence Detection System using SYBR Green PCR Master Mix (Applied Biosystems, Austin, TX, USA). SOS1 (salt overly sensitive 1) and NHX1 (Na^+^/H^+^ exchanger 1) primers were developed from *C. quinoa* using methods previously described by Maughan et al. [45] and Morales et al. [46], respectively. The pairs of primers utilized for real-time PCR are presented in Table S2. The *GAPDH* gene [47] was used to normalize expression data and as a housekeeping gene for the estimation of the relative transcript levels of genes of interest in each comparative analysis.

### 2.4. Statistical Analysis

All data are represented as the mean ± SD of three replicates. Two-way analysis of variance (ANOVA) was used to test the hypothesis that the genotype and salinity concentration affect the studied characteristics of plants. If there were significant differences between the means, comparisons among different groups were performed using Duncan’s multiple range tests [48]. *p* values ≤ 0.01 were considered to be statistically significant for all statistical tests. Data and statistical analysis were carried out using Excel 2016 and Minitab V.19. Principal component analysis (PCA) was conducted to study the morphological relationships among the studied quinoa genotypes using PAST software [49]. A cluster dendrogram and matrix plot of the studied genotypes was created based on all the study data (morpho-physiological traits and unique bands generated from molecular markers). Additionally, Pearson correlation among the studied genotypes and all revealed data was computed using PAST software [49]. The heatmap was used to study the similarity and dissimilarity among studied taxa based on morphological traits using the TBtools package [50]. Cluster phylogeny of genotypes based on molecular markers was conducted using dendrogram construction using the unweighted pair group method of averages (UPGMA) in NTSYSpc software version 2.1 [51] and was used for dendrogram construction using the unweighted pair group method of averages (UPGMA).

## 3. Results

### 3.1. Genetic Diversity Analysis

#### 3.1.1. ISSR and SCoT Marker Characterization

Ten primers were utilized to study the genetic diversity among the studied quinoa genotypes using ISSR molecular markers, generating 176 total amplicons with molecular sizes ranging from 80 to 1430 bp; the ISSR pattern is illustrated in Figure S1. Total polymorphic amplicons from this marker were 160; the greatest number of polymorphic amplicons (27) was produced from ISSR 1, and the least polymorphic amplicons (10) were produced from ISSR 3 and ISSR 10. The highest polymorphism percentage (100%) was generated from ISSR 6 and ISSR 10. The highest PIC was observed in ISSR 8 with a value of 0.88, and the lowest value was 0.6 seen in ISSR 7 (Table 2).

For the SCoT marker, ten primers were used to generate 156 total amplicons with 133 polymorphic amplicons and sizes ranging from 132 to 1900 bp; the SCoT pattern is shown in Figure S2. The greatest number of polymorphic amplicons (26) was generated from SCoT 7, and the least polymorphic amplicons (three) were from ScoT 3 and SCoT 10. The highest polymorphism (100%) was produced from SCoT 2, 3, 7 and 8, and PIC values generated from all primers varied significantly from 0.84 in SCoT 2 to 0.32 in SCoT 10 (Table 2). The greatest number of positive and negative unique amplicons generated from ISSR characteristics for LINE-6 were 28 and three fragments, respectively, whereas the greatest number of positive, unique amplicons from SCoT characteristics for M-28 was 16 fragments. ISSR marker produced the highest polymorphism percentage with a value of 90.91% (Table 3). Based on the molecular markers ISSR and SCoT, a cluster dendrogram using UPGMA correlation coefficient was created and is presented in Figure S3. The studied genotypes were classified into two clusters: the first involved LINE-6 genotype with a similarity of 87%, whereas the second involved the other genotypes. The second cluster was classified into two sub-clusters: one included M-28 genotype with a similarity of 78%, while the second included Q-37, S-10, and Regeolone-3 genotypes. Similarity correlation among the studied genotypes based on molecular markers is demonstrated in Table S3, which indicates that the highest similarity was seen between S-10, and Regeolone-3 genotypes with a value of 0.657, whereas the lowest similarity was seen between M-28 and LINE-6 genotypes with a value of 0.44. 

#### 3.1.2. Protein Pattern (SDS-PAGE)

**Figure 1:**
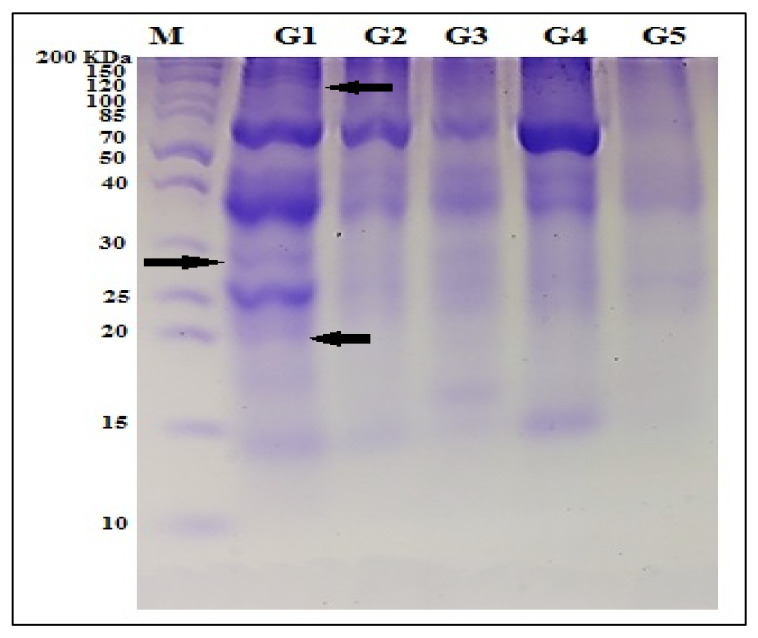
Protein SDS-PAGE profile for the studied Quinoa genotypes, G1: M-28; G2: Q-37; G3: S-10; G4: Regeolone-3; G5: LINE-6; arrows: unique bands for M-28 genotype.

Eleven total protein bands were recorded in quinoa genotypes with molecular weights varying from 12 to 200 KDa, with three polymorphic bands detected as unique characteristic bands which distinguished the M-28 genotype. The polymorphism percentage among the studied quinoa was 27.27% (Table 3). The protein profile is illustrated in Figure 1.

### 3.2. Overall Performance of Morpho-Physiological Traits under Salinity Stress

Five quinoa genotypes were grown under four different conditions: 60, 80, 120, or 160 mM NaCl was added. Analysis of variance revealed significant variations between the studied salinity concentrations for different genotypes. In this regard, the highest and lowest values for all studied characteristics in both seasons were obtained for the 60 and 160 mM NaCl treatments, respectively; however, sodium (Na) and proline contents revealed the opposite relationship to NaCl treatment (data not shown). In the same trend, ANOVA revealed significant differences between the studied genotypes at the various salinity treatments. M-28 genotype was found to have the highest values for all the studied traits, while LINE-6 genotype represented the lowest in both seasons (data not shown).

**Figure 2:**
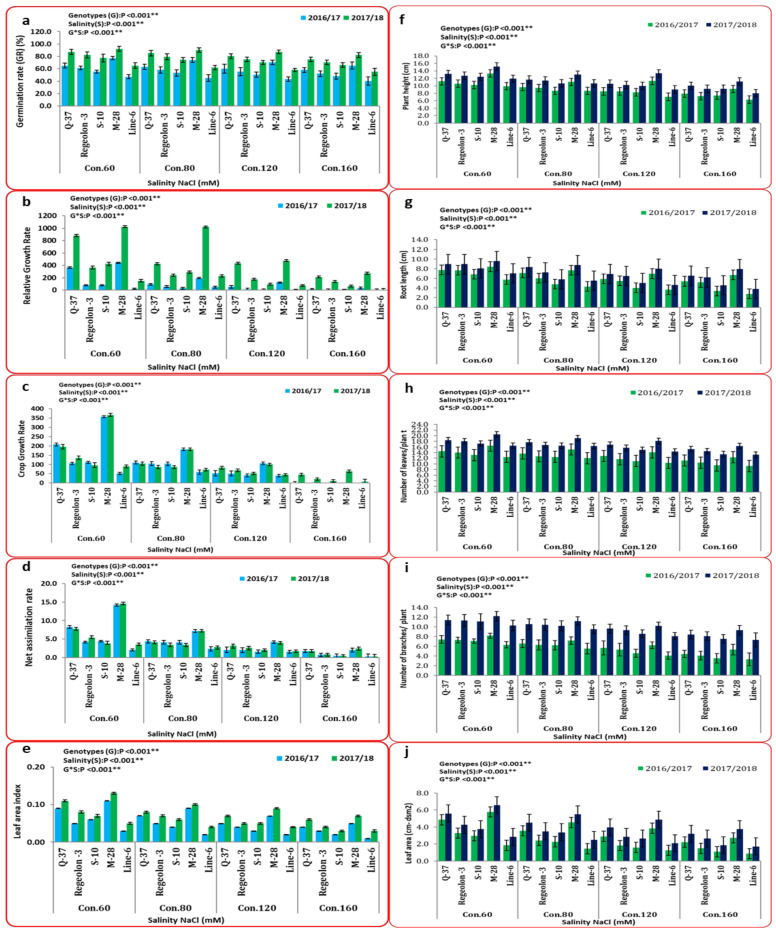
Mean performance (combined data of two seasons) represented as (mean ± SD) for (**a**) germination rate, Growth traits: (**b**) relative growth rate, (**c**) crop growth rate, (**d**) net assimilation rate, physiological traits: (**e**) leaf area index, (**f**) plant height (cm), (**g**) root length (cm), (**h**) number of leaves/plants, (**i**) number of branches/plants, and (**j**) leaf area. All data are means of three replicates. Two-way ANOVA was performed to determine the effect of different quinoa genotypes on the studied traits. For all statistical tests, *p* values ≤ 0.01 ** were considered highly statistically significant.at 0.01% probability level.

On the other hand, ANOVA for germination analysis, growth analysis, yield, components, and chemical composition parameters revealed that the interactions between treatments and genotypes were significant at *p* ≤ 0.01. The data presented in Figure 2 indicate that the highest values for germination analysis (77.27 and 92.18) were obtained by M-28 genotype under 60 mM NaCl in the 1st and 2nd growing seasons, respectively. Meanwhile, the lowest values (40.35 and 55.27) were exhibited by LINE-6 genotypes under 160 mM NaCl in the 1st and 2nd growing seasons, respectively. Likewise, the M-28 genotype recorded the highest values for growth traits, including plant height (13.31 and 15.16 cm), root length (8.41 and 9.58 cm), number of leaves/plant (16.44 and 20.44 cm), number of branches/plant (8.22 and 12.22), and leaf area (5.77 and 6.56 cm), for the 1st and 2nd growing seasons, respectively, under 60 mM NaCl treatment. Furthermore, the LINE-6 genotype presented the lowest values for the same traits (6.30 and 8.04 cm, 2.79 and 3.79 cm, 9.22 and 13.22, 3.33 and 7.33, and 0.86 and 1.76 cm) for the 1st and 2nd growing seasons, respectively, under 160 mM NaCl treatment. Figure 2 demonstrates that the highest growth analysis trait (RGR, CGR, NAR, and LAI) values (14.18 and 14.58 g/g/day; 356.67 and 366.57 g/g/day.cm^2^; 442.1 and 1025 gm^−2^ day^−1^; and 0.11 and 0.13) for the 1st and 2nd growing seasons, respectively were consistently obtained by M-28 genotype under 60 mM NaCl treatment. Additionally, LINE-6 genotype attained the lowest values for RGR (0.31 and 0.30 g/g/day), CGR (7.86 and 7.61 g/g/day·cm^2^), NAR (0.42 and 48 gm^−2^ day^−1^), and LAI (0.01 and 0.03) under 160 mM NaCl treatment in both seasons.

**Figure 3:**
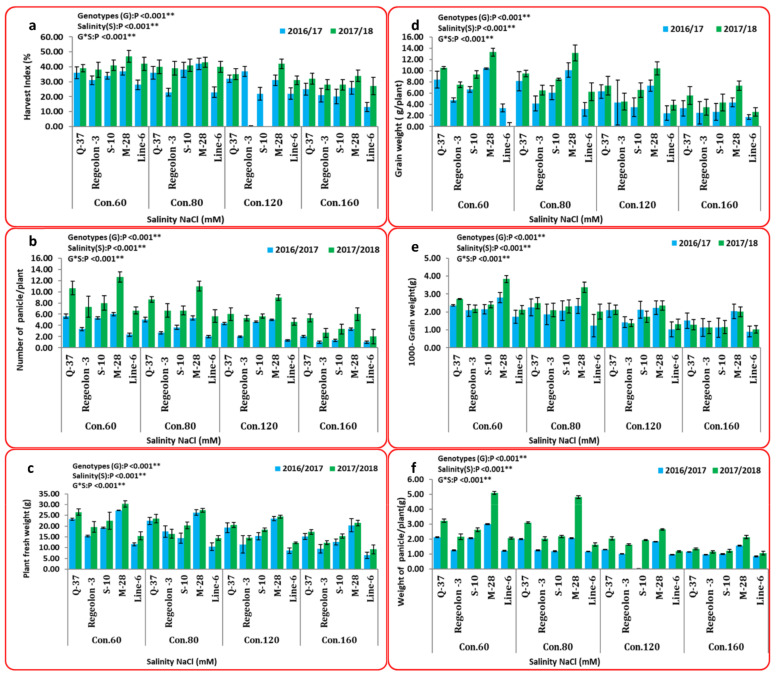
Mean performance (combined data of two seasons) represented as (mean ± SD) for yield and its component traits: (**a**) harvest index (HI; %), (**b**) number of panicles/plants, (**c**) plant fresh weight (g), (**d**) grain weight (g/plant), (**e**)1000-grain weight (g) and (**f**) weight of panicles/plant (g). All data are means of three replicates. Two-way ANOVA was performed to test the effect of different quinoa genotypes on the studied traits. For all statistical tests, *p* values ≤ 0.01 ** were considered highly statistically significant.at 0.01% probability level.

The results presented in Figure 3 indicate that the maximum values for grain weight (10.35 and 13.29 g), harvest index (37.0% and 47.0%), 1000-grain weight (2.80 and 3.84 g), number of panicles per plant (6.00 and 12.66), and weight of panicles/plant (3.00 and 5.09 g) were obtained by M-28 genotype under 60 mM NaCl treatment in both seasons. On the other hand, the minimum values (1.7 and 2.63 g; 13.0% and 27.0%; 0.91 and 1.03 g; 1.00 and 2.00; and 0.84 and 1.07 g) of the studied traits were detected in LINE-6 genotype under 160 mM NaCl. In the same context, the M-28 genotype recorded the highest value for plant fresh weight (30.37 g), while the LINE-6 genotype with 160 mM NaCl treatment attained the lowest value (9.22 g). 

**Figure 4:**
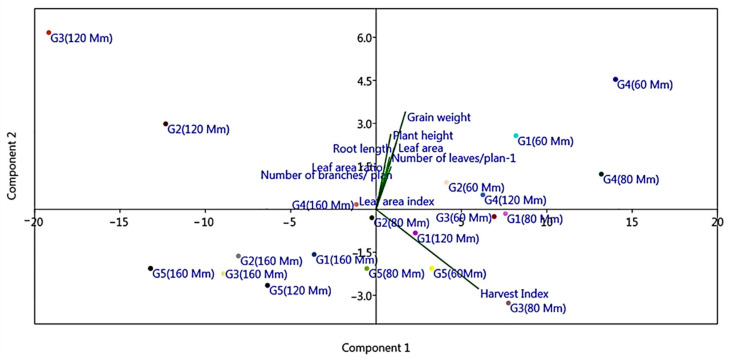
Principal component analysis (PCA) of the quantitative morphological traits for the studied treated quinoa genotypes. The first component demonstrated variance of 91.228% and the second component demonstrated variance of 6.877%; G1: M-28; G2: Q-37; G3: S-10; G4: Regeolone-3; G5: LINE-6.

Principle component analysis (PCA) indicating morphological trait variation among the studied genotypes is presented in Figure 4. The first and second components of PCA revealed a total variation of 91.228% and 6.877%, respectively, with a total value of 98.105%. The axes indicated that the most significant traits which can vary among genotypes were harvest index (HI), grain weight (GW), and plant height (PH). 

**Figure 5:**
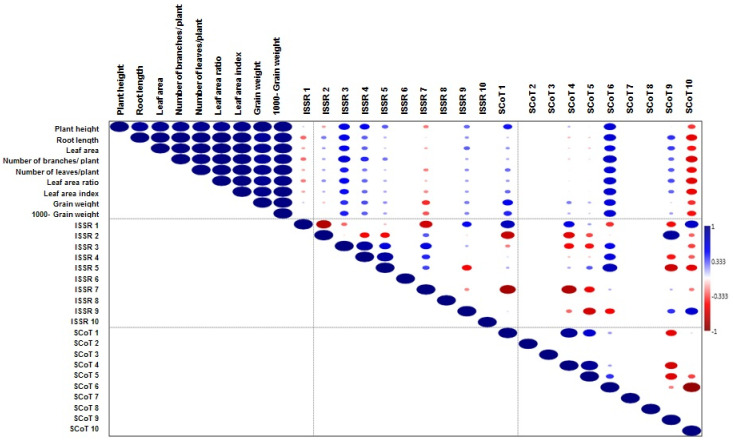
Pearson Correlation among some (morpho-physiological traits & molecular attributes). The blue color indicates a positive correlation, and the red color indicates a negative correlation. The size of the circle, proportional to the correlation coefficient and intensity of color, represented the magnitude of the value.

Due to the importance of ISSR and SCoT data in distinguishing the studied genotypes, the relationships between the studied morpho-physiological traits and the unique bands generated from molecular markers were further analyzed. A cluster dendrogram was generated using the UPGMA correlation coefficient, which classified the studied genotypes into two clusters: one contained LINE-6 genotype separated from the other genotypes, and the second cluster was further divided into two sub-clusters, with one containing genotypes M-28 and S-10 and the second containing genotypes Q-37 and Regeolone-3 genotypes. This result confirmed the previous finding that genotype LINE-6 is the least salt tolerant of the studied genotypes (Figure S4). To predict and identify the specific potential unique bands that might be responsible for salinity tolerance in the studied genotypes, a Pearson correlation done among some of the essential studied traits under salinity stress and all the unique bands generated from the studied genotypes (Figure 5). The results clearly stated that the unique bands generated from ISSR3 and SCoT6 primers had a high correlation with all the studied traits under salinity stress. From those unique bands, two bands were generated from the M28 genotype, and two bands were generated from the LINE6 genotype. Oppositely, there was a negative correlation between SCoT 10 and all the selected morphological traits. These bands might be considered helpful markers linked to salinity tolerance or sensitivity in quinoa breeding programmers however, further studies aimed at purification, sequencing and analysis of these bands might be necessary in future work.

### 3.3. Biochemical Analysis under Salinity Stress

**Figure 6:**
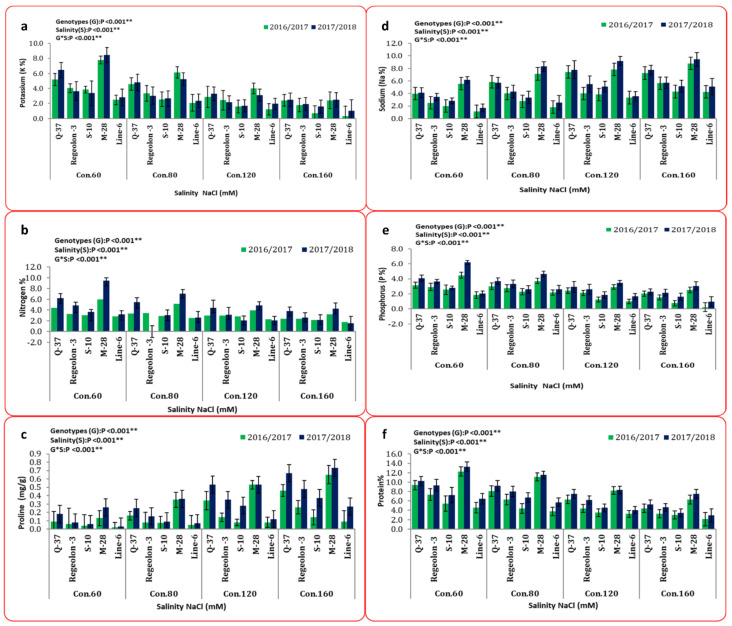
Mean performance (combined data of two seasons) represented as (mean ± SD) for biochemicals traits: (**a**) potassium, (**b**) Nitrogen, (**c**) proline, (**d**) sodium, (**e**) phosphorus, and (**f**) protein. All data are means of three replicates. Two-way ANOVA was performed to test the effect of different quinoa genotypes on the studied traits. For all statistical tests, *p* values ≤ 0.01 ** were considered highly statistically significant.at 0.01% probability level.

The chemical composition results presented in Figure 6 indicate significant differences between salinity and genotypes. The highest recorded means for K (7.80% and 8.45%), P (4.46% and 6.18%), N (6.01% and 9.47%), and protein (12.25% and 13.28%) contents in the 1st and 2nd growing season, respectively, were achieved by M-28 genotype under 60 mM NaCl. On the other hand, LINE-6 genotype under 160 mM NaCl treatment demonstrated the lowest means (0.33% and 1.05%; 0.22% and 0.97%; 1.77% and 1.51%; 2.10% and 2.88%) for K, P, N, and protein content, respectively in both seasons. Additionally, the maximum values for Na (8.80% and 9.44%) and proline (0.65% and 0.73%) were achieved by the M-28 genotype with 160 mM NaCl treatment in both seasons. However, the minimum values for Na (1.12% and 9.44%) and proline (0.02% and 0.03%) were found in LINE-6 genotype with 60 mM NaCl treatment in both seasons.

**Figure 7:**
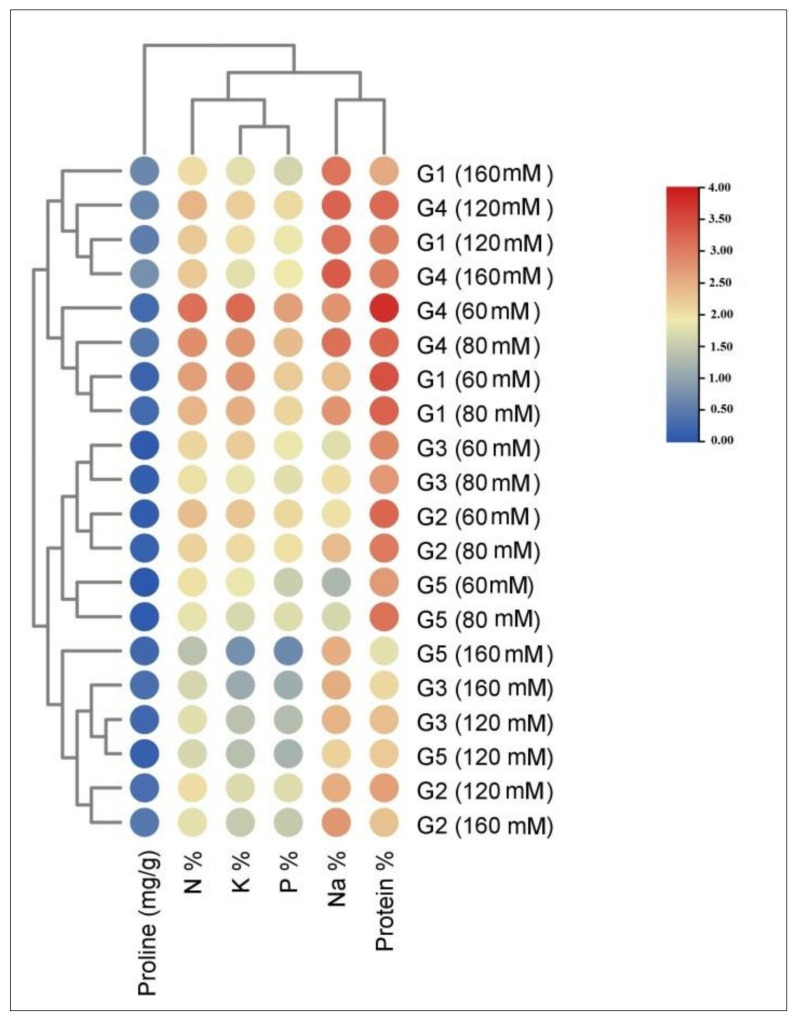
Heat map based on elements and phytochemical compounds of quinoa genotypes treated with different concentrations of NaCl; G1: M-28; G2: Q-37; G3: S-10; G4: Regeolone-3; G5: Line -6.

The heat map presented in Figure 7 shows the variations in elements and phytochemicals between the studied quinoa genotypes under salinity stress. The treated genotypes were separated into two groups: the first included M-28 and Regeolone-3 genotypes treated with different concentrations of NaCl, while the second included the other three genotypes (Q-37, S-10, and LINE-6) also treated with different concentrations of NaCl. The red color indicates high similarity between the studied treatments, while blue indicates low similarity. 

### 3.4. Gene Expression Analysis under Salinity Stress

**Figure 8:**
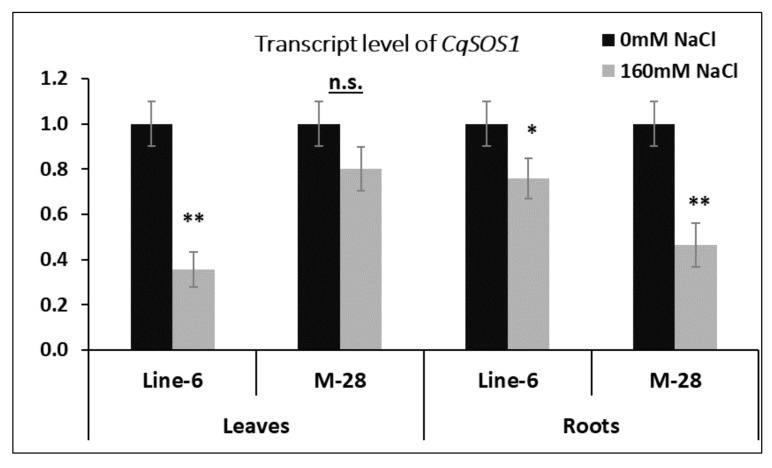
Expression analysis of *CqSOS1*. Data represent leaves and roots of two quinoa genotypes. Twelve-day-old seedlings were gradually treated with 160 mM NaCl over six days. Results are presented as the mean fold-change in relative expression over the control from three biological and technical replicates, normalized to *GAPDH* (reference) gene expression. Bars represent standard deviation. *, ** Significant and highly significant at 0.01% probability level.

Analysis of *CqSOS1* and *CqNHX1* gene expression was conducted for M-28 genotype (tolerant) and LINE-6 genotype (sensitive) quinoa treated with 160 mM NaCl, using qPCR to quantify the role of each salt-coping mechanism. Figure 8 presents the transcript level of *CqSOS1* in treated genotypes compared with untreated ones. Interestingly, our results demonstrate that the expression of *CqSOS1* in both studied genotypes and tissues was downregulated compared with that seen in nontreated seedlings.

**Figure 9:**
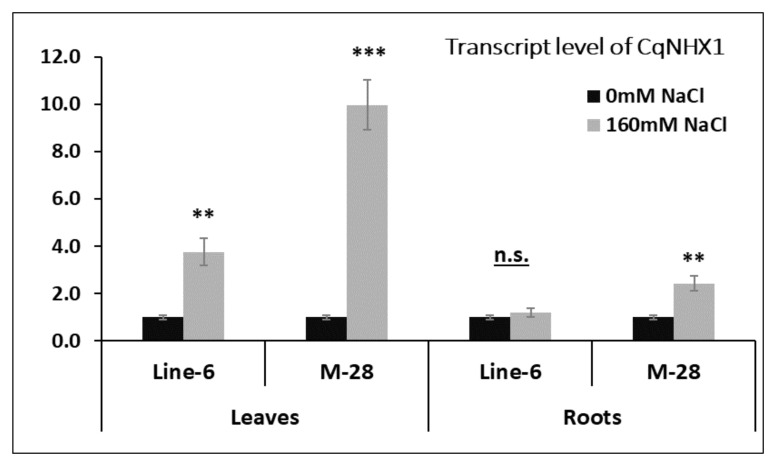
Expression analysis of *CqNHX1*.Data represent leaves and roots of two quinoa genotypes. Twelve-day-old seedlings were gradually treated with 160 mM NaCl over six days. Results are presented as the mean fold-change in relative expression over the control from three biological and technical replicates, normalized to *GAPDH* (reference) gene expression. Bars represent standard deviation. **, *** Significant and highly significant at 0.01% probability level.

Moreover, the reduction percentage of the roots of the LINE-6 genotype was lower than that of the M-28 genotype. Meanwhile, the reduction percentage of the leaves demonstrated the opposite trend. In addition, the *CqNHX1* transcript level was investigated in treated genotypes and is compared with that of untreated genotypes in Figure 9. mRNA transcript level was higher in leaves than in roots. It was clear that leaf expression sharply increased 10-fold for the tolerant genotype (M-28), while the sensitive genotype (LINE-6) increased just 2-fold, compared with untreated samples. In a similar trend, root samples exhibited upregulation in both genotypes, but the expression in the tolerant genotype was 1.5 times higher than that of the sensitive genotype.

**Figure 10:**
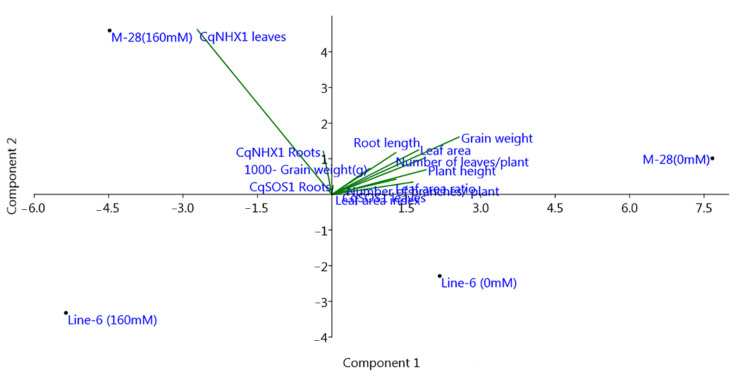
Principal component analysis (PCA) of some morpho-physiological traits and relative gene expressions for M-28 and Line-6 genotypes under salinity stress. The first component demonstrated variance of 74.155% and the second component demonstrated variance of 20.521%.

To clarify correlation between relative gene expressions SOS1 and NHX1 genes and morpho-physiological traits Principal Component Analysis (PCA) was computed for M-28 and Line-6 quinoa genotypes under salinity stress (Figure 10). The first two principal components (PC1 &PC2) with Eginvalue greater than 2.6 and explained total variance 74.155% and 20.521% respectively. The eigenvalues are considered the best measure for the quality of ordination and of the strength of the genotypes–morpho-physiological relationship. Results from PCA indicated that relative gene expression of NHX1 in leaves was the most significant one, followed by grain weight, leaf area and plant height. Length of the arrow refers to the most powerful for NHX1 gene variable and the direction of the arrow points refers to highest morpho-physiological traits change.

## 4. Discussion

A genetic variability is an important tool for discerning information regarding the adaptation of species and cultivars to biotic and abiotic conditions, which lead to changes in the genetic composition of these plants. Many molecular markers have been used to estimate the genetic diversity of quinoa in several investigations [13,14,52]. ISSR markers are a valuable genetic tool for assessing the relationships among quinoa genotypes [53,54]. In this investigation, ISSR markers generated 176 total bands with 68 positive unique bands and four negative bands; these unique bands were used for species and genotype identification [55,56]. ISSR markers were also used to detect genetic variations in quinoa [57]. The polymorphism percentage revealed by ISSR primers was 90.91% higher than both polymorphism percentages produced by the same marker in Al-Naggar et al. [58] and Saad-Allah and Youssef [59]’s studies, which reported values of 61.83% and 31.47%, respectively. Polymorphism information content (PIC) in this study varied from the lowest value of 0.32 (revealed by SCoT) to the highest value of 0.88 (displayed by ISSR). The value of PIC ranges from 0 to 1, with values closer to 1 indicating higher polymorphism. Additionally, PIC is classified as high informative (PIC > 0.5), moderate informative (0.25 < PIC < 0.5), and low informative (PIC < 0. 25) [60]. The value of PIC in this study was higher than both the values of PIC, ranging from 0.10–0.25, revealed by the SRAP marker for 135 quinoa accessions in Thailand [61], and the mean PIC value produced using SRAP of 0.59 for 32 quinoa genotypes in Egypt [62]. Concerning polymorphism, the ISSR marker was superior and more efficient than the SCoT marker in this study, with ISSR detecting 90.91% while SCoT detected 85.26%. Similar results were obtained by Abd El-Moneim [63] and Gowayed and Abd El-Moneim [64] for wheat and by El-Mansy et al. [65] for tomato. On the other hand, Abdein et al. [66,67] revealed that SCoT showed more polymorphism than ISSR in tomato and squash, respectively. A cluster dendrogram based on molecular markers divided the studied genotypes into two clusters. Genotype M-28 in one cluster and LINE-6 in the other cluster, which is in agreement with similarity indices and provides the lowest similarity between M-28 and LINE-6 genotypes.

Quinoa contains main protein fractions from albumins and globulins. The globulins (chenopodin) are called 221 11S-type, which include two subunits: acidic subunits, with a molecular weight range of 30–40 KDa, and basic subunits, with a molecular weight range of 20–25 KDa. The proteins in the lower protein band, with a molecular weight range of 8–11 KDa, are called 2S-type proteins [10,22,23,68]. The present study revealed that the SDS-PAGE technique exhibited protein band alterations among the studied quinoa genotypes. Genotype M-28 had three unique bands with molecular weights of 20, 25, and 29 KDa; this differentiation is based on the difference in intensities and molecular weights of polypeptides that are the final product of the transcription and translation process [69]. Quinoa genotypes had a low level of protein polymorphism of 27.27%; this low percentage may be due to the protein-conserving nature of seeds [70,71].

Several authors have documented that quinoa genotypes exhibit significant genetic diversity in agro-physiological reactions when grown under saline conditions [72,73]. In the same trend, in this research, all of the studied traits were significantly affected by different artificial salinity levels. Figure 2, Figure 3 and Figure 6 demonstrated that all of the studied parameters decreased—except for Na and proline levels—with increasing salinity. Furthermore, the variation between the studied genotypes in response to salinity was distinct.

The most sensitive phase for plants, including halophytes, is germination [74]. In most quinoa genotypes, concentrations of 100 to 250 mM NaCl have no effect on germination rates [75]. Nevertheless, germination is delayed at doses of 150 to 250 mM NaCl [76]. In this study, reduced germination under salt stress conditions may have been due to the osmotic potential. Similar results were revealed by [77]. Growth parameters and leaf area of the investigated genotypes were impacted by salinity stress, primarily through a decrease in the photosynthesis rate, an imbalance between the photosynthesis and respiration rates of the entire plant or decreased water uptake. This result is in line with Saleem et al. [78] and Long [79]. The altered and/or reduced supply of certain plant nutrients might cause a lower growth rate in certain genotypes, resulting in less relative growth. Similar results were observed by Talebnejad et al. [80] for NAR and Islam et al. [81] for CGR and RGR. On the other hand, Riccardi et al. [82] demonstrated that RGR and NAR were not significantly different between saline- and nonsaline-treated plants.

Plant height is one of the most sensitive traits affected by salt stress, according to Jacobsen et al. [83]. In this investigation, plant height was reduced, potentially due to the toxic impact of the NaCl utilized, unequal nutrient uptake, or reduced cell division and DNA replication in interphase. Similar findings were reported by Arshadullah et al. [84] and Hussain et al. [85].

Clearly, salinity reduced grain weight/plant for M-28 and LINE-6 genotypes from (13.29 and 6.26) at 60 mM NaCl to (7.30 g and 2.63 g) at 160 mM NaCl, respectively. These results agreed, too, with those of Hussain et al. [85]. In the same context, the obtained results clarified that the lowest values of grain yield, 1000-grain weight, and harvest index were recorded for 160 mM NaCl, while the highest values were recorded for the 60 mM NaCl treatment. These results were in agreement with Miranda et al. [86], Hirich et al. [87], and Algosaibi et al. [88]. Also, the decrease in the number and size of the panicles/plant has been related to lower seed yield [87]. This phenomenon was also observed in our study. Koyro and Eisa [89] demonstrated that plant fresh weights were all significantly reduced in the presence of salinity.

On the other hand, the highest recorded means for K^+^ contents and Na^+^ were achieved by the M-28 genotype, while the lowest recorded means were obtained for the LINE-6 genotype. Similarly, Orsini et al. [90] reported that Na^+^ was induced by 150–750 mM NaCl in Chilean cv. BO78. Because quinoa plants collect Na^+^, which is readily available for cytosolic osmotic adjustment and maintaining turgor pressure, the increased Na^+^ absorption should be followed by accelerated K^+^ transport from root to shoot to maintain an appropriate K^+^:Na^+^ ratio in leaves [91]. Our data demonstrated that Na^+^ increased dramatically with increasing salinity in the leaves of the M-28 genotype, with a rapid spike in Na^+^ concentration occurring only at 160 mM, and a similar trend was seen in genotype LINE-6 genotype. In genotype M-28, the K^+^ concentration in leaves dropped with rising salinity levels at 160 mM, whereas it increased significantly in genotype M-28. Additionally, genotype M-28 was shown to be superior to genotype LINE-6 in terms of producing greater dry weight at the maximum salinity level, which was associated with greater leaf K^+^ accumulation than in genotype LINE-6. This result is in line with Saleem et al. [78]. The enhanced absorption of K^+^ by the roots at increasing Na^+^ levels may explain the M-28 genotype’s resilience to salt stress. One of the most essential physiological indications for salt tolerance is proline accumulation [92]. A significant increase in proline content was detected under 160 mM in leaves of the M-28 genotype. This result was in line with the findings of other researchers, including Prado et al. [93] and Derbali et al. [94].

Overall, all tested genotypes could survive under the highest salinity stress (160 mM NaCl). Additionally, genotype M-28 was tolerant and the least affected, while genotype LINE-6 was sensitive and, following salinity treatment, its agro-physiological characteristics were drastically affected.

Plants undergo a variety of changes in response to abiotic stress, ranging from physiological adaptation to gene expression. Several species’ pivotal genes associated with Na^+^ transport have been cloned, and their role in salt tolerance has been examined [95]. In this study, both studied genotypes (tolerant and sensitive) exhibited a lower transcript abundance of *CqSOS1* under 160 mM NaCl, which disagrees with the acclimation role of the trait encoded by this gene. Our results agree with the finding of Maughan et al. [45], which states that saline treatment caused no significant expression of *CqSOS1A* and *CqSOS1B* genes in roots. This observation would suggest that this treatment did not induce Na^+^ exclusion at the root level. On the contrary, in the absence of salinity, *CqSOS1A* and *CqSOS1B* were expressed more strongly in roots than in leaves; however, saline treatment produced an upregulation of both genes in leaves, indicating that cytoplasmic Na^+^ was migrating out of the roots [45].

Under salt or osmotic stress, NHX proteins may provide protection by compartmentalizing K and Na in the vacuole, thereby preventing toxic Na/K ratios in the cytosol. Overexpression of the NHX family of Na^+^/H^+^ antiporters in diverse plant species has resulted in enhanced salt tolerance in many instances [96,97]. In this regard, the obtained results of *CqNHX1* expression were consistent with those of Ruiz et al. [75], who found that 300 mM NaCl increased *CqNHX1* expression in the tolerant cultivar but not in the salt-sensitive cultivar, implying that compartmentation in the vacuole was not used in the latter. These results confirm our hypothesis regarding tolerance mechanisms in the studied genotypes. Moreover, detected differences in expression levels may indicate a preventative response rather than initiating expression upon stress. These results agreed with Ruiz et al. [75], who conducted a comparative analysis of three genes (*CqNHX1*, *CqSOS1A*, and *CqSOS1B*) and found that saline conditions produced greater increments in *CqNHX1* than in *CqSOS1B*, with even less in *CqSOS1A*.

The combined morpho-physiological and molecular analysis is a target for breeding programs to enhance crop yield under drought stress [98] and salinity stress [64]. Salt stress led to morphological, physiological, biochemical, and molecular changes that adversely affected the studied genotype’s growth and productivity. To completely comprehend the negative effects of high salinity in plants, several combined evaluations are required [99]. Significant interactions among genotype and irrigation conditions for several agronomical variables indicate the genotypic flexibility available to the species and the need to evaluate genotypic performance under each growing condition [100]. It is worth mentioning that the morpho-physiological and biochemical results proved that the M28 genotype is more tolerant than the LINE-6 genotype. This fact is linked to the higher level of *CqNHX1* gene expression in the M28 genotype than in the LINE-6 genotype. In addition, the highest and lowest means of K^+^ and Na^+^ contents were recorded for the M28 genotype and LINE-6 genotype, respectively, indicating that vacuolar Na^+^ or K^+^ compartmentation is an important tolerance mechanism in the tolerant genotype and compartmentation in the vacuole was less active in the sensitive genotype. In the same way, these genotypes exhibited a wide range of genetic variability, with LINE-6 genotype generating more unique positive and negative bands (50 bands) than M28 genotype (42 bands) using ISSR and SCoT markers. These markers could have further potential in genotyping and revealing polymorphisms directly related to gene function.

## 5. Conclusions

The present study reinforces the utility of morpho-physiological, biochemical, and molecular analysis to select quinoa genotypes with variable performance under different salinity conditions. There was an association based on morpho-physiological and molecular data, which indicates that both types of characterization (phenotype and genotype) are essential for understanding the differentiation between quinoa genotypes. Moreover, the high genetic diversity found by SCoT, ISSR, and protein analysis could be exploited in breeding programs to obtain new cultivars and provide relevant information for diversity conservation. Moreover, there was a wide range of variability in all morpho-physiological and biochemical traits. There was upregulation of *CqNHX1* and downregulation of *CqSOS1* genes in leaf or root tissues of the studied genotypes. Our results revealed M-28 (tolerant) and Line-6 (sensitive) genotypes.

## Figures and Tables

**Table 1:** Name and origin of the studied genotype’s.

Genotype Name	Source
M-28	Denmark
Q-37	Chile
S-10	Denmark
Regeolone-3	Chile
LINE-6	Denmark

**Table 2:** Features of ISSR and SCoT molecular markers used to study the genetic diversity of quinoa genotypes.

Molecular Markers		Amplicon Size (bp) Range (bp)	Total No. of Amplicons	Monomorphic Amplicons	Polymorphic Amplicons	Unique Amplicons	% Polymorphism	PIC
ISS	ISSR 1	196–870	29	2	27	17	93.1	0.81
	ISSR 2	182–660	18	2	16	6	88.89	0.76
	ISSR 3	215–965	11	1	10	4	90.91	0.76
	ISSR 4	185–845	19	2	17	4	89.47	0.77
	ISSR 5	200–1180	19	1	18	8	94.74	0.81
	ISSR 6	145–750	17	0	17	4	100	0.86
	ISSR 7	95–970	20	5	15	8	75	0.6
	ISSR 8	185–925	15	0	15	4	100	0.88
	ISSR 9	80–1430	18	3	15	7	83.33	0.68
	ISSR 10	275–1155	10	0	10	2	100	0.77
	Average	-	32	2.91	29.09	11.64	83.30%	0.77
SCoT	SCoT 1	215–1030	14	2	12	5	85.71	0.76
	SCoT 2	154–1464	20	0	20	5	100	0.84
	SCoT 3	175–840	21	0	21	4	100	0.83
	SCoT 4	300–1240	15	1	14	6	93.33	0.81
	SCoT 5	155–588	10	5	5	2	50	0.33
	SCoT 6	150–790	8	3	3	2	37.5	0.35
	SCoT 7	195–1400	26	0	26	5	100	0.82
	SCoT 8	132–1900	25	0	25	8	100	0.82
	SCoT 9	190–305	9	5	4	0	44.44	0.35
	SCoT 10	225–735	8	5	3	2	37.5	0.32
	Average	-	15.6	2.1	13.3	3.8	74.85%	0.62

PIC: polymorphism information content.

**Table 3:** Comparison between features generated from molecular markers and protein SDS-PAGE for the studied Quinoa genotypes.

Features		Molecular Markers		SDS-PAGE Protein
		ISSR	SCoT	
**Band size range**		80–1430 bp	132–1900 bp	12–200 KDa
**Total bands**		176	156	11
**Polymorphic bands**		160	133	3
**Unique bands**	**Positive**	19 (M-28), 5(Q-37), 8 (S-10), 4 (Regeolone-3), 28(LINE-6)	19 (M-28), 1(Q-37), 2 (S-10), 16 (LINE-6)	3 (M-28)
	**Negative**	1(M-28), 3(LINE-6)	3 (M-28), 3 (LINE-6)	-
**% Polymorphism**		90.91%	85.26%	27.27%

## Data Availability

Relevant data applicable to this research are within the paper.

## Supplementary Materials

The following are available online at https://www.mdpi.com/2223-7747/10/12/2802/s1, Figure S1. Amplification products generated from ten ISSR primers. Figure S2. Amplification products generated from ten SCoT primers. Figure S3. Cluster Dendrogram of the studied quinoa genotypes based on UPGMA analysis using the similarity matrix generated by ISSR and SCoT markers. Figure S4. Cluster Dendrogram of the studied quinoa genotypes based on UPGMA analysis using the similarity matrix generated from morpho-physiological traits and molecular attributes (ISSR &SCoT). Table S1. Sequences of ISSR and SCoT primers used in studying genetic diversity of quinoa. Table S2. Primer information used for gene expression analysis in *Chenopodium quinoa*. Table S3. Similarity correlation among selected genotypes of quinoa.
