# Supplementary material for: Quinoa (Chenopodium quinoa Willd.): Genetic Diversity According to ISSR and SCoT Markers, Relative Gene Expression, and Morpho-Physiological Variation under Salinity Stress
Source: Plants (Basel). 2021 Dec 17;10(12):2802. doi: 10.3390/plants10122802 (PMC8707205; doi:10.3390/plants10122802)
Supplement: Supplementary file 1 [file plants-10-02802-s001.zip › plants-1472782-supplementary.pdf]

Table S1. Sequences of ISSR and SCoT primers used in studying genetic diversity of quinoa.

	Markers	Sequences 5′–3′	Annealing Temperature ºC
**ISSR**	ISSR 1	(CT) 8 GC	**55**
	ISSR 2	(GA)6CC	
	ISSR 3	(CAC)3GC	
	ISSR 4	(GTG)3GC	
	ISSR 5	CCA(CT)8	
	ISSR 6	(CT)8G	
	ISSR 7	(AG)8G	
	ISSR 8	(AG)8C	
	ISSR 9	(GA)8T	
	ISSR 10	GAG(CAA)5	
**SCoT**	SCoT 1	CAATGGCTACCACTAGCC	**50**
	SCoT 2	CAATGGCTACCACTAACG	
	SCoT 3	CAATGGCTACCACTAGCG	
	SCoT 4	ACAATGGCTACCACTAGG	
	SCoT 5	ACAATGGCTACCACTACC	
	SCoT 6	ACAATGGCTACCACCATC	
	SCoT 7	CCATGGCTACCACTAGCA	
	SCoT 8	CCATGGCTACCACTAGCG	
	SCoT 9	CAACAATGGCTACCACCG	
	SCoT 10	CCATGGCTACCACCGCCC	

Table S2. Primer information used for gene expression analysis in *Chenopodium quinoa*.

Gene	F	Length	R	Length
**SOS1**	CCTCATGATGCTTCCGACAA	**20**	CCGAGTCAAGTGCTTCATCA	**20**
**NHX1**	ATCAGTTTACGAGGTCAGGGCACA	**24**	GAGGCTTTGTCAGCAACCCAAACA	**24**
**GAPDH**	GGTTACAGTCATTCAGACACCATCA	**25**	AACAAAGGGAGCCAAGCAGTT	**21**

Table S3. Similarity correlation among selected genotypes of quinoa.

**Genotypes**	M-28	Q-37	S-10	Regeolone-3	Line -6
M-28	1				
Q-37	0.602	1			
S-10	0.497	0.617	1		
Regeolone-3	0.467	0.563	0.657	1	
Line -6	0.44	0.476	0.497	0.593	1

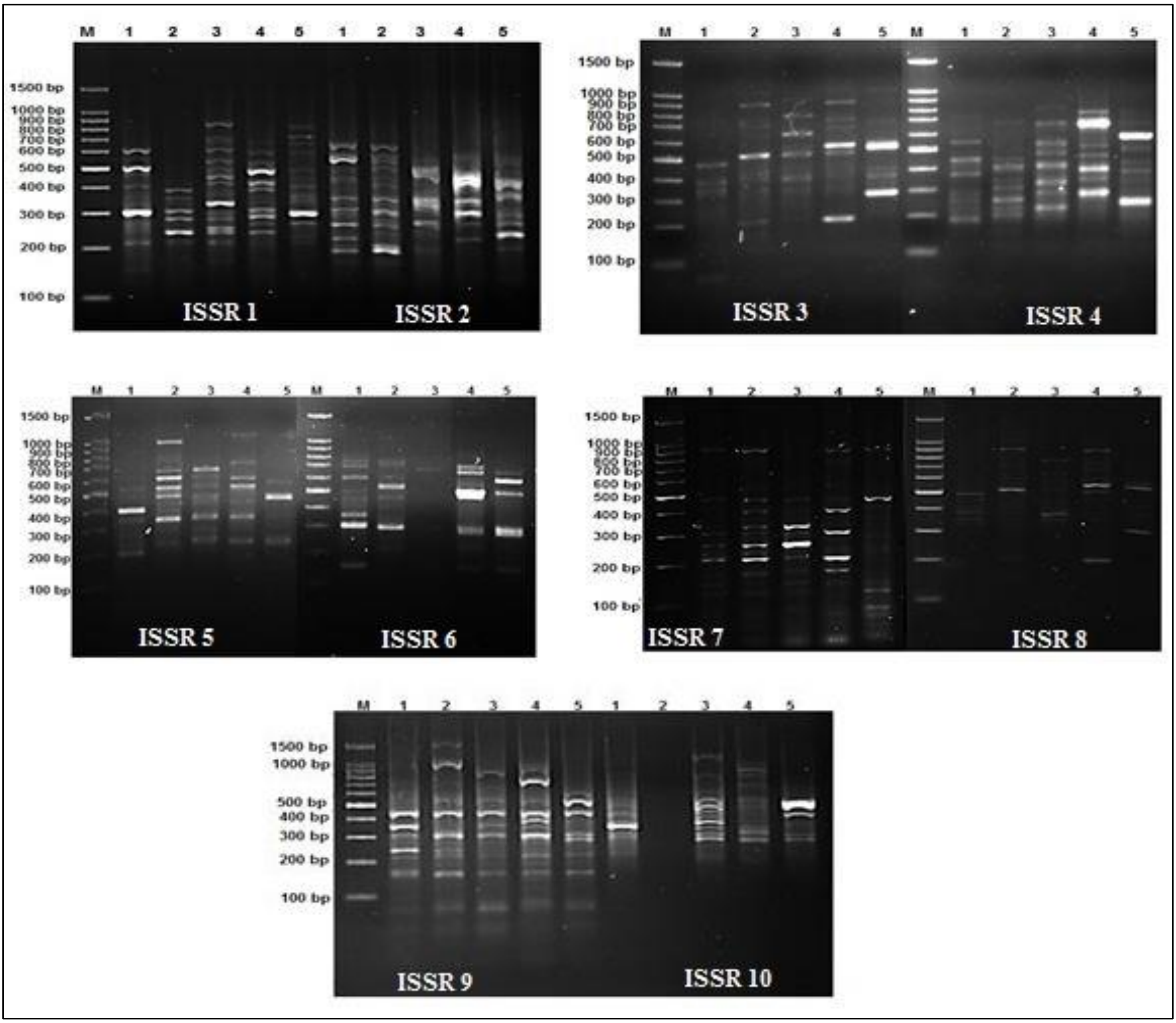

Figure S1. Amplification products generated from ten ISSR primers, 1= M-28, 2= Q-37, 3= S-10, 4= Regeolone-3, 5= Line -6.

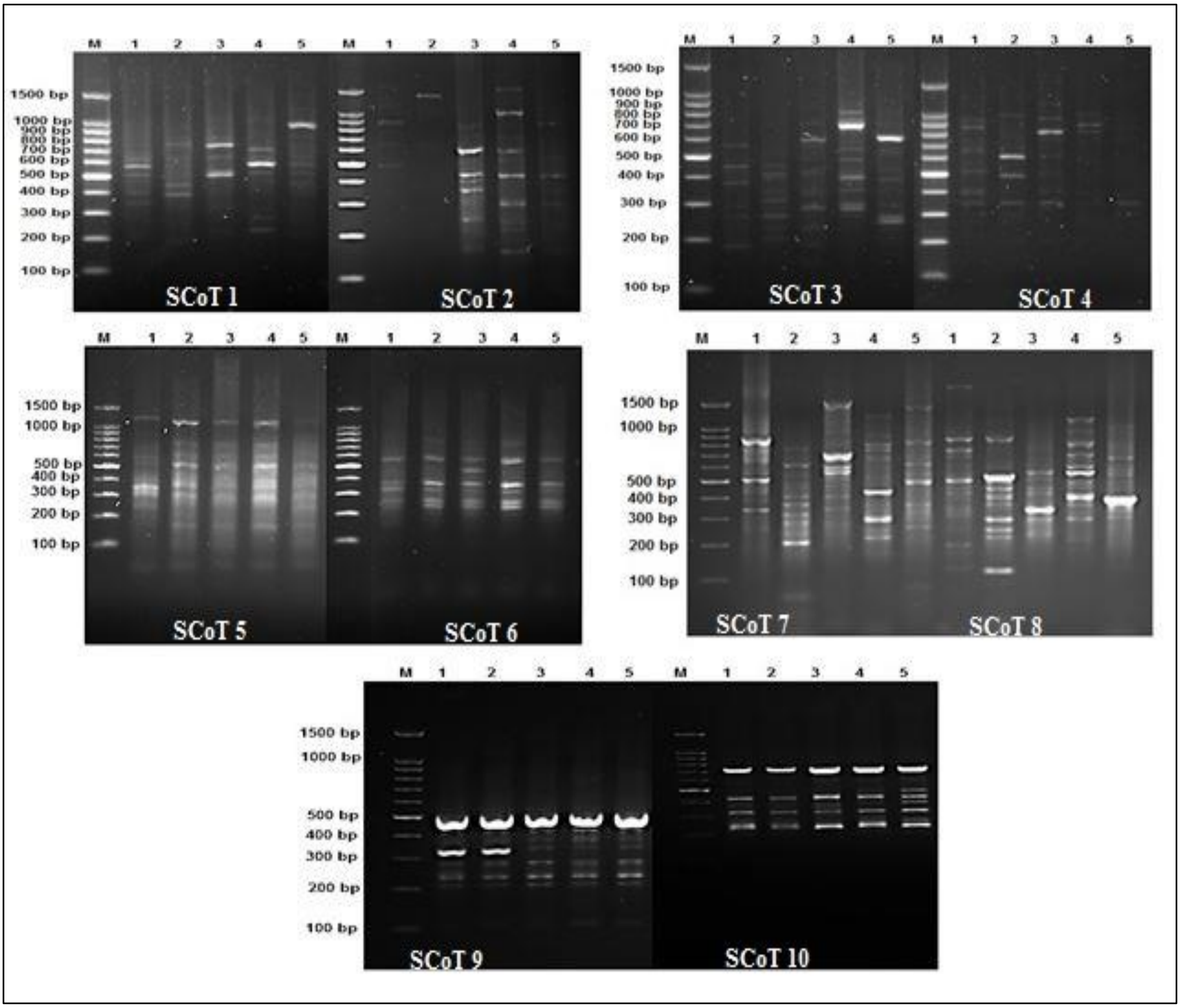

Figure S2. Amplification products generated from ten SCoT primers, 1= M-28, 2= Q-37, 3= S-10, 4= Regeolone-3, 5= Line -6.

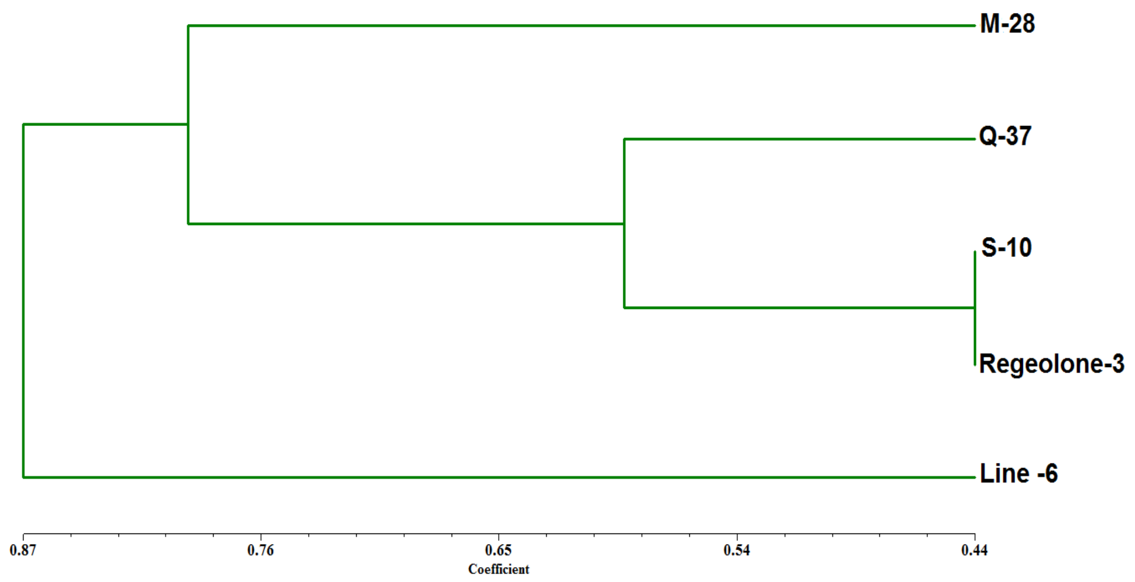

Figure S3. Cluster Dendrogram of the studied quinoa genotypes based on UPGMA analysis using the similarity matrix generated by ISSR and SCoT markers.

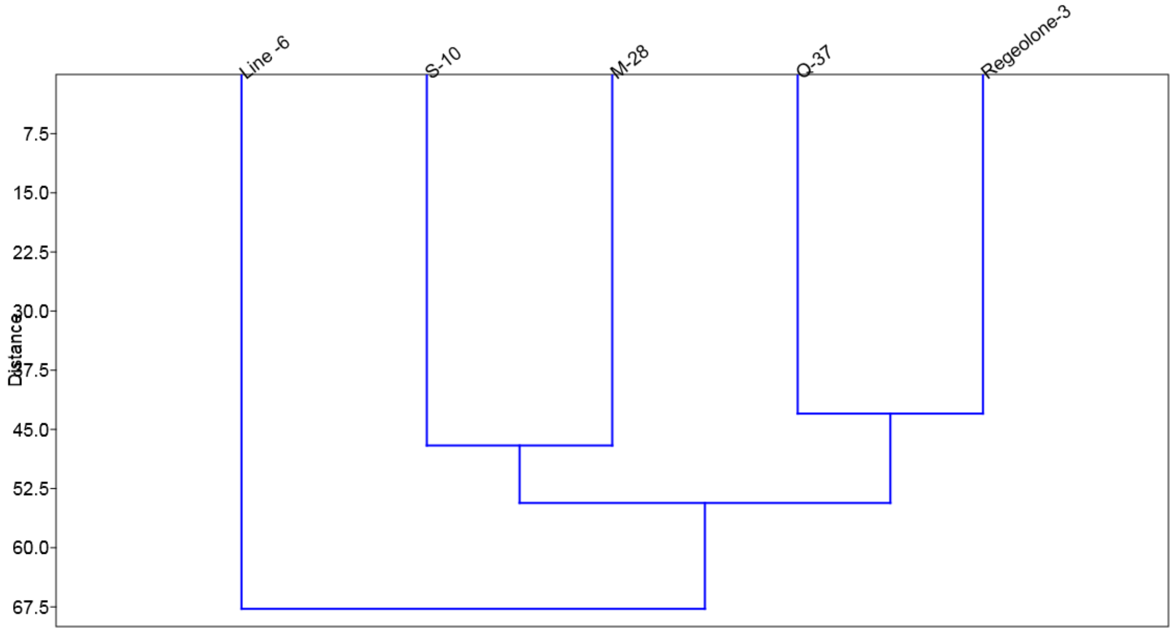

Figure S4 Cluster Dendrogram of the studied quinoa genotypes based on UPGMA analysis using the similarity matrix generated from morpho-physiological traits and molecular attributes (ISSR &SCoT).
